# Delineating Molecular Regulatory of Flavonoids Indicated by Transcriptomic and Metabolomics Analysis during Flower Development in *Chrysanthemum morifolium ‘Boju’*

**DOI:** 10.3390/ijms251910261

**Published:** 2024-09-24

**Authors:** Zhuannan Chu, Rui Xiong, Xingxing Peng, Guangsheng Cui, Ling Dong, Weiwen Li

**Affiliations:** Key Laboratory of Horticultural Crop Germplasm Innovation and Utilization (Co-Construction by Ministry and Province), Institute of Horticulture, Anhui Academy of Agricultural Sciences, Hefei 230001, China; chuzhnan1988@163.com (Z.C.); m15255173358@163.com (R.X.); 15212447331@163.com (X.P.); cuigs8@163.com (G.C.); dlaaas@163.com (L.D.)

**Keywords:** *Chrysanthemum morifolium* ‘*Boju*’, flavonoid metabolism, kaempferol

## Abstract

Flavonoids are pharmacologically active compounds in flowers of *Chrysanthemum morifolium* ‘*Boju*’ (*C. morifolium*); however, the molecular regulatory network governing flower development remains largely elusive. Flower samples were collected at four stages, namely budding (BD), bud breaking (BB), early blooming (EB), and full blooming (FB), for omics analysis. We revealed distinct transcriptional regulation patterns at these four stages of the flower from the perspective of differentially expressed unigenes (DEGs). There are 152 DEGs shared among the three comparative groups (BD vs. BB, BB vs EB, EB vs FB), wherein the expression of 44 DEGs (including AtADT6, MDL3, and ROMT) continues to be upregulated, and 85 DEGs (including CYP81E, TPS-Cin-1, and TPS-Cin-2) showed persistent downregulation with flower development. Flavonoid-targeted metabolomics identified 118 differentially abundant metabolites (DAMs) in the FB group compared to the BD stage; the top three upregulated and downregulated metabolites are Cyanidin-3-O-(6″-O-malonyl)glucoside-5-O-glucoside, Luteolin-7-O-(6″-caffeoyl)rhamnoside, Kaempferol-3-O-(6″-p-coumaroyl)glucoside and Chrysoeriol-6,8-di-C-glucoside-7-O-glucoside, Kaempferol, Kaempferol-3,7-O-dirhamnoside, respectively. These DAMs were predominantly enriched in “flavonoid biosynthesis”, “isoflavonoid biosynthesis”, and “flavone and flavonol biosynthesis” pathways. AtADT6, MDL3, ROMT, CYP81E, TPS-Cin-1, and TPS-Cin-2 were correlated with kaempferol. Our findings provide a new idea for interfering with flavonoid production, especially kaempferol, in flowers.

## 1. Introduction

*Chrysanthemum morifolium* ‘*Boju*’ (*C. morifolium*) is a kind of medicinal chrysanthemum Boju, a plant used in traditional Chinese medicine, and is one of the five major medicinal chrysanthemums stipulated in the 2020 edition of the “Chinese Pharmacopoeia” [1]. Pharmacopoeia defines the medicinal chrysanthemum with indicators such as flavonoids, chlorogenic acid, luteoloside, and 3,5-O-dicaffeoylquinic acid. The medicinal chrysanthemum primary therapeutic effects include dispersing wind, clearing heat, soothing the liver, and brightening the eyes, with additional benefits of heat-clearing and detoxifying properties [2]. Medicinal chrysanthemum is employed in the treatment of wind-heat-type colds, headaches, dizziness, red and swollen eyes, blurred vision, as well as various skin disorders and toxic swellings, which is largely attributed to its antioxidant, anti-inflammatory, antimicrobial, and anti-genotoxic properties [3,4]. However, the specific genetic networks and metabolic pathways through which *C. morifolium* exerts its pharmacological effects remain unclear.

Flavonoids are the primary pharmacologically active compounds in flowers of *C. morifolium* [5]. Flavonoids, characterized by the C6-C3-C6 structure, play not only crucial roles in various aspects of plant physiology, including growth, development, flowering, and fruiting, as well as antimicrobial and disease resistance processes but also possess potent functions of anti-oxidant, anti-mutagenic, anti-inflammatory, and anti-carcinogenic [6]. Flavonoids are subdivided into six subgroups, including flavones, flavonols, flavanones, flavanonols, flavanols or catechins, anthocyanins, and chalcones [6]. Currently, research and development activities related to flavonoid compounds primarily involve the isolation, identification, characterization, and functionality of flavonoids, as well as their applications for health benefits. Little is known about the metabolic regulatory networks of flavonoids in *C*. *morifolium*. According to a previous study., the key genes involved in flavonoid metabolism identified in *C*. *morifolium* are currently limited to flavonoid 3′-hydroxylase (F3′H), flavonoid 3′,5′-hydroxylase (F3′5′H), and cytochrome P450 [7]. Wang et al. verified that flavonoids are associated with genes of chalcone isomerase (CHI), F3H, dihydroflavonol-4-reductase (DFR), and anthocyanidin synthase (ANS) [8]. This knowledge gap severely constrains advancements in both industrial and agricultural efforts aimed at enhancing the production of active flavonoid components in *C*. *morifolium*. Moreover, flavonoids are the largest class of compounds in the polyphenol class, and the starting substrates for flavonoid synthesis in plants are coumaroyl coenzyme A and malonyl coenzyme A from the phenylpropane metabolic pathway. Firstly, phenylalanine generates cinnamic acid under the action of phenylalanine ammonia-lyase (PAL), which is further hydroxylated under the action of cinnamic acid 4-hydroxylase (C4H) to convert into coumaric acid and then forms coumaroyl coenzyme A under the catalytic action of 4-coumaric acid coenzyme A ligase (4CL), and coumaroyl coenzyme A and malonyl coenzyme A is catalyzed by chalcone synthase (CHS) (this enzyme catalyzes the generation of chalcone skeleton of all flavonoids) to generate chalcone followed by isomerization of chalcone isomerase (CHI) to produce colorless naringenin, naringenin as the main metabolite into the synthesis pathway of other flavonoids [9]. Although the synthesis pathway of flavonoid compounds is conserved in plants, many enzymes alter the basic backbone of flavonoids to produce different kinds of flavonoid compounds due to different external factors, such as environmental stresses [10]. Thus, differences in the metabolism of flavonoid compounds between species may be an evolutionary feature of plants. In particular, the phenylpropanoid pathway, one of the most important sources of metabolites associated with environmental responses, is thought to provide a high degree of evolutionary diversity for land plant-specific adaptations [11,12]. The study of natural variations in the composition and content of flavonoids and gene regulation in *C*. *morifolium* not only contributes to the understanding of the function of flavonoids in *C*. *morifolium* flowers but also provides new metabolic evidence for the bio-evolutionary characteristics of *C*. *morifolium*. Therefore, the exploration and elucidation of the regulatory network of flavonoid metabolism in *C*. *morifolium* are urgent and imperative.

Transcriptomic and metabolomic co-analysis offers a comprehensive approach to unraveling the regulatory network underlying the metabolism of pharmacologically active compounds in medicinal plants. By integrating information from both transcriptomes and metabolomes, researchers gain a more holistic understanding of the molecular mechanisms governing the synthesis, modification, and regulation of bioactive components in medicinal plants. This integrated approach provides insights into gene expression patterns and metabolic changes, allowing for the identification of key pathways and regulatory nodes involved in the biosynthesis and modulation of pharmacologically active compounds. Therefore, the combined analysis of transcriptomic and metabolomic data provides a powerful strategy to uncover the regulatory networks and metabolic pathways involved in the synthesis and modulation of pharmacologically active compounds in medicinal plants. Currently, several studies have employed a combined analysis of transcriptome and metabolome to elucidate the physiological processes in various Asteraceae plants. For instance, Yu et al. reveal the characteristics of volatile oils in *Chrysanthemum morifolium* [13], Zhu et al. demonstrate the molecular mechanism underlying variation in floral scent during flower development of *Chrysanthemum indicum* var. *aromaticum* [14], Zou et al. reveal redirection of the phenylpropanoid metabolic flux in different colored *Chrysanthemum morifolium* [15]. Moreover, Li et al. found that low-nitrogen conditions improve the accumulation of flavonoids in snow chrysanthemum using integration analysis of physiological, transcriptomic, and metabolomics [16]. However, as of now, there are no multi-omics studies revealing the regulatory network of flavonoid metabolism at different developmental stages of flowers of *C*. *morifolium*.

We aim to employ a combined analysis of the transcriptome and flavonoid-targeted metabolome to unveil the gene expression and metabolic changes of genes and flavonoid compounds during the four developmental stages of *C*. *morifolium* flowers. This endeavor seeks to provide preliminary insights into the molecular regulatory network of flavonoids.

## 2. Results

### 2.1. Transcriptomic Landscape of Flower of C. morifolium at Different Four Stages

**Figure 1:**
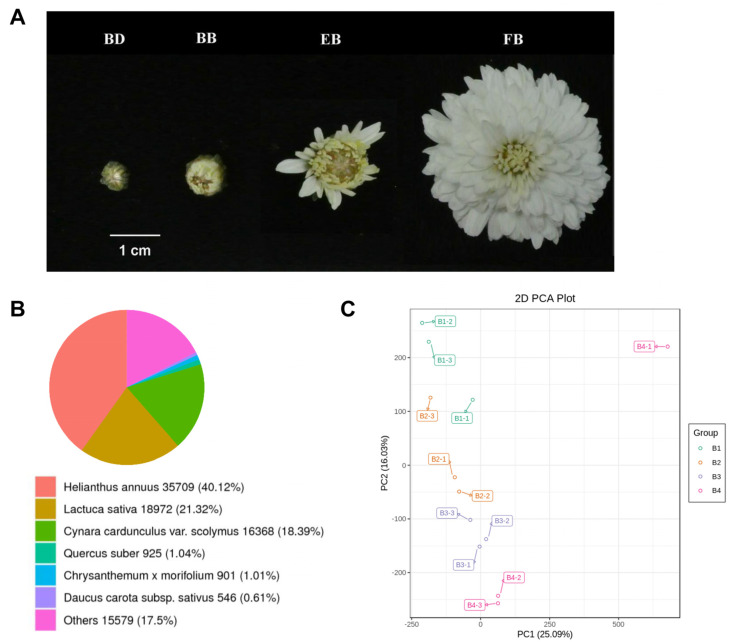
Transcriptomic profiles of four developmental stages of C. *morifolium* flower. (**A**) Representative images of C. *morifolium* flowers at four different stages of development. (**B**) Statistical pie charts of transcript sequences mapped to different species in the NR database. (**C**) Principal component analysis plot of all samples in the transcriptome. B1, B2, B3, and B4 means BD, BB, EB, and FB, respectively.

To characterize the gene expression patterns during different developmental stages of *C*. *morifolium* flowers, we collected flower samples from four stages for transcriptome sequencing. Representative images of flowers from the four stages (BD, BB, EB, FB) are shown in Figure 1A. In the present study, a total of 102.86 Gb clean data were obtained, with each sample yielding approximately 7 Gb of clean data, the Q30 base percentage exceeded 91% for all samples, and the GC content for each sample was around 42% (Supplementary Table S1). These results preliminarily indicate that the transcriptome sequencing data are of high quality and suitable for further analysis. Furthermore, after quality control, the assembly of clean reads resulted in a total of 796,142 transcripts and 223,237 unigenes, with N90 values of 294 bp and 315 bp, respectively (Supplementary Table S2). By comparing against the NR database, we examined the similarity of the obtained transcript sequences with those of closely related species and obtained functional information on homologous sequences. The results are depicted in Figure 1B, with *Helianthus annuus* (35,709, 40.12%) having the highest score, followed by *Lactuca sativa* (18,972, 21.2%) and *Cynara cardunculus* var. *scolymus* (16,368, 18.39%). In comparison, there are only 2.65% of the aligned sequences from the *Quercus suber* (1.04%), *Chrysanthemum x morifolium* (1.01%), and *Daucus carota* subsp. *Sativus* (0.61%). Moreover, we employed principal component analysis to characterize the differential transcriptomic profiles among the four stages of samples. The results revealed a total separation rate of 41.12% among the four groups, with samples from each stage distinctly separated (Figure 1C), indicating significant transcriptomic differences at different flower developmental stages.

### 2.2. Flowers of C. morifolium Exhibit Differential Transcriptomic Profiles at Different Four Stages

**Figure 2:**
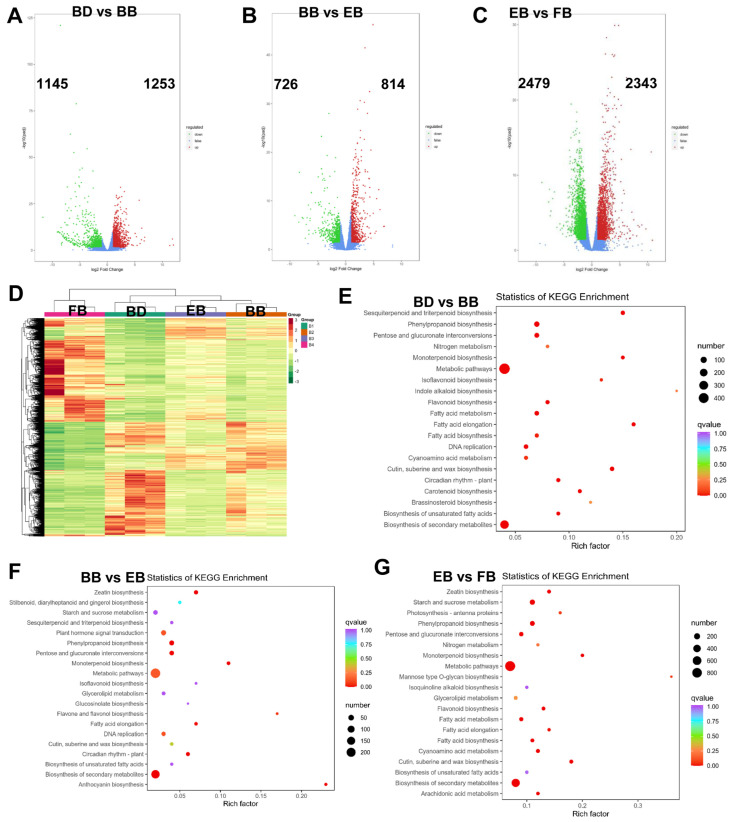
Flowers of C. *morifolium* exhibit differential transcriptomic profiles at four different stages. Volcano map of differentially expressed unigenes in the comparison groups of BD vs. BB (**A**), BB vs. EB (**B**), BB vs. FB (**C**). Red indicates up-regulated genes, green indicates down-regulated genes, and blue indicates non-significant genes. (**D**) The hierarchical clustering heatmap of all differentially expressed unigenes. Bubble plot of KEGG enrichment of differentially expressed unigenes in the comparison groups of BD vs. BB (**E**), BB vs. EB (**F**), BB vs. FB (**G**).

To investigate whether there is differential expression of specific genes regulating the production of medicinal compounds within the flowers of *C*. *morifolium* at four developmental stages, we conducted differential expression analysis of the unigenes in flowers at these four stages. Compared to the BD stage, 2398 unigenes (1253 up-regulated and 1145 down-regulated) were differentially expressed during the BB stage (Figure 2A). In comparison to the BB stage, there were 1,540 differentially expressed unigenes (814 up-regulated and 726 down-regulated) within the flowers during the EB stage (Figure 2B). Additionally, 4822 unigenes (2343 up-regulated and 2479 down-regulated) were found to be differentially expressed in the FB stage compared to the EB stage (Figure 2C). The hierarchical clustering heatmap results indicated that these differentially expressed unigenes clustered the samples into four distinct categories (Figure 2D), suggesting that there are distinct transcriptional regulations occurring at the four stages of flower development.

Next, to explore the signaling pathways primarily involved in the differential expression of these unigenes leading to the production of pharmacologically active compounds in flowers, we conducted a KEGG pathway analysis. Interestingly, we observed that the differentially expressed unigenes generated from the three comparative groups across the four stages collectively participate in the pathways of “fatty acid elongation”, “monoterpenoid biosynthesis”, “pentose and glucuronate interconversions”, “cutin, suberine, and wax biosynthesis”, “biosynthesis of unsaturated fatty acids”, “biosynthesis of secondary metabolites” (Figure 2E–G). Additionally, the “flavonoid biosynthesis” pathway was significantly enriched in the differentially expressed genes of both the BD vs BB and EB vs FB comparative groups (Figure 2E,G). These results suggest that the differentially expressed unigenes across the four stages persistently activate the aforementioned pathways, which may be associated with the production of pharmacologically active compounds in the flowers of *C*. *morifolium*.

### 2.3. Identification of Common Differentially Expressed Unigenes

**Figure 3:**
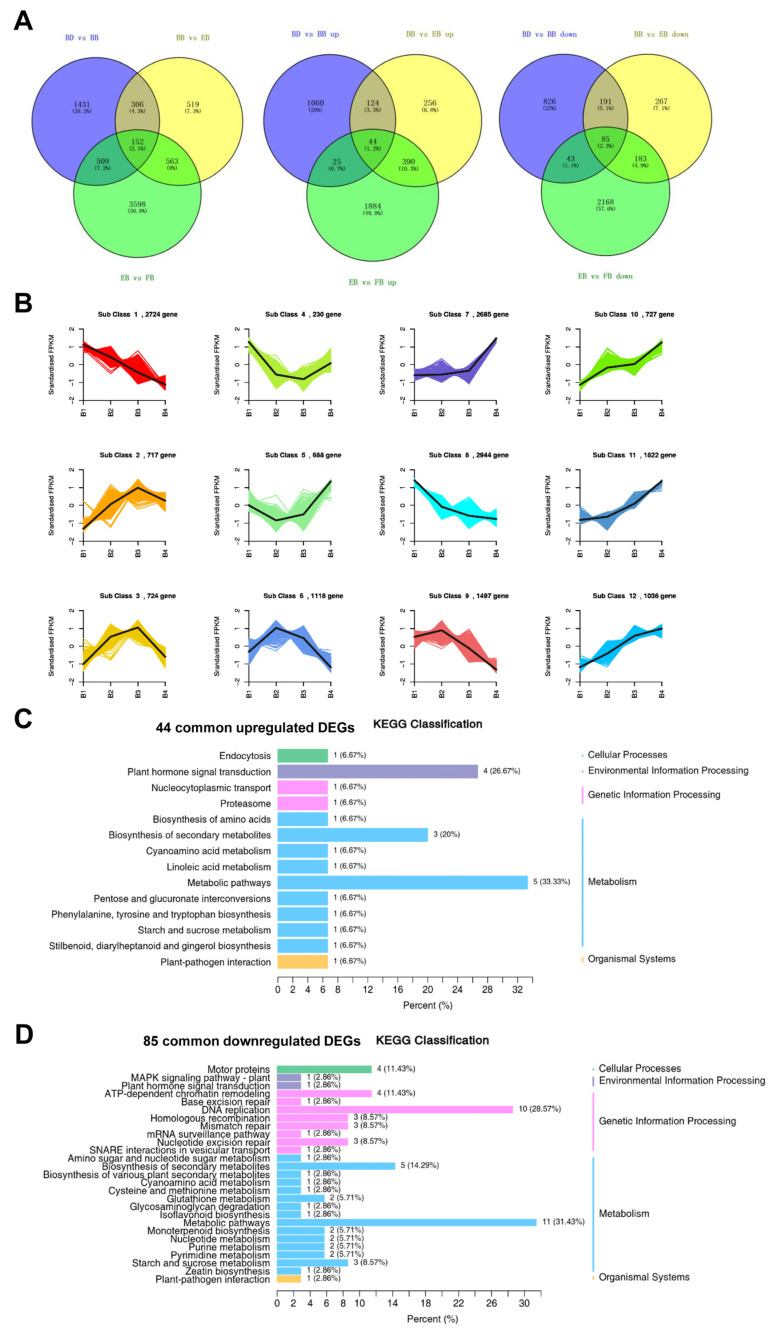
Identification of common differentially expressed unigenes in all the comparison groups. (**A**) A Venn diagram of intersected differentially expressed unigenes from the three comparative groups. (**B**) Kmeans_clustering analysis of differentially expressed unigenes in four groups. (**C**) Bubble plot of KEGG enrichment of 44 common upregulated differentially expressed unigenes. (**D**) Bubble plot of KEGG enrichment of 85 common downregulated differentially expressed unigenes.

To further elucidate the continually differentially expressed unigenes along the developmental trajectory of the flower of *C. morifolium*, we intersected the lists of differentially expressed unigenes from the three comparative groups. The results indicate that there are 152 unigenes shared among the three comparative groups, wherein the expression of 44 unigenes continues to be upregulated with flower development, exhibiting a trend similar to Class 10, 11, and 12 in kmeans_cluster, while 85 genes showed persistent downregulation, following a trend akin to Class 1 and 8 in kmeans_cluster (Figure 3A,B). Interestingly, we found that 3 (including Arogenate dehydratase/prephenate dehydratase 6, chloroplastic [EC:4.2.1.91 4.2.1.51] (AtADT6), (R)-mandelonitrile lyase [EC:4.1.2.10] (MDL3), and trans-resveratrol di-O-methyltransferase [EC:2.1.1.240] (ROMT)) of these 44 consistently upregulated differentially expressed unigenes were enriched in the Biosynthesis of secondary metabolites and one ROMT was involved in the Stilbenoid, diarylheptanoid and gingerol biosynthesis pathway (Figure 3C). In addition, most of the 85 shared unigenes were involved in metabolism-related pathways; among them, Cytochrome P450 81E8 ([EC:1.14.14.90 1.14.14.89] CYP81E) is involved in Isoflavonoid biosynthesis 1,8-cineole synthase [EC:4.2.3.108] (TPS-Cin-1) and TPS-Cin-2 are involved in Monoterpenoid biosynthesis (Figure 3D). Given that these six genes (AtADT6, MDL3, ROMT, CYP81E, TPS-Cin-1, and TPS-Cin-2) are persistently differentially expressed during flower development and participate in flavonoid-related pathways, we hypothesized that they may be key regulatory genes for flavonoid production during flower development.

### 2.4. Quality Control of the Flavonoid-Targeted Metabolome across the Four Stages of Flower Development

To characterize the flavonoid metabolism in the flowers of C. *morifolium* across four developmental stages, we conducted a flavonoid-targeted metabolomics analysis. Total ions current of UPLC-MS/MC of quality control samples showed clear peaks both in positive and negative modes (Supplementary Figure S1A), and clear peaks were also observed in MRM detection of multimodal maps (Supplementary Figure S1B), indicating that the system was able to quantify metabolites. Moreover, the overlapped total ions current maps demonstrated excellent reproducibility, indicating the high stability of the instrument (Supplementary Figure S1C). This ensures crucial assurance for the repeatability and reliability of the data. In this study, utilizing the UPLC-MS/MS detection platform and a custom-built database, a total of 248 metabolites were detected (Supplementary Table S3). Additionally, principal component analysis revealed that the samples from the four groups shared 72.65% of the variation based on 248 metabolites, with PC1 and PC2 contributing 53.28% and 19.37%, respectively (Supplementary Figure S1D), indicating significant differences in metabolites across different stages of flowers.

### 2.5. Metabonomics Landscape of Flavonoid Compounds in the Flowers Undergoes Significant Changes, Particularly in the Early and Mature Stages

**Figure 4:**
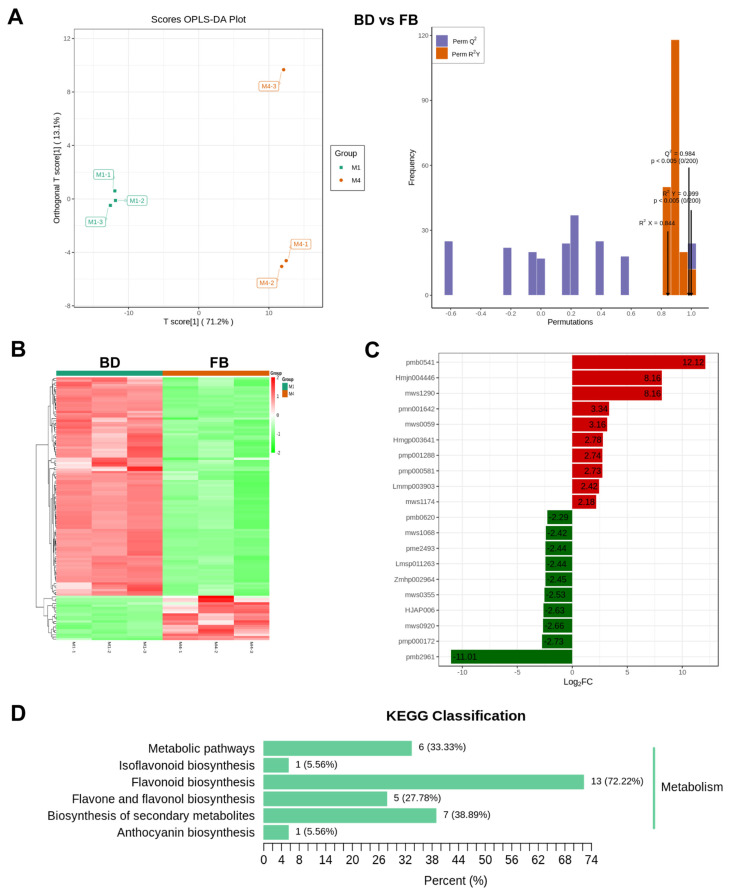
The metabolic landscape of flavonoid compounds in the flowers undergoes significant changes, particularly in the early and mature stages. (**A**) Scores of OPLS-DA plot and OPLS-DA model in the comparison groups of BD vs. FB. M1, M2, M3, and M4 means BD, BB, EB, and FB, respectively. (**B**) The hierarchical clustering heatmap of differentially abundance metabolites between BD vs. FB. (**C**) Histogram of the top 20 differentially abundance metabolites between BD vs. FB. (**D**) Histogram of KEGG enrichment pathway of differentially abundance metabolites between BD vs. FB.

Subsequently, we identified differential metabolites at different stages of flower development. As shown in Supplementary Table S4, compared to the BD stage, there were 45 significant DAMs in the flowers at the BB stage, while the metabolites remained nearly unchanged between the BB and early EB stages. However, in comparison to the EB stage, there were 32 DAMs in the flowers at the FB stage. All these inter-group differences were supported by the OPLS-DA model (Supplementary Figure S2). Additionally, OPLS-DA revealed an 83.3% variation between early BD flowers and mature FB flowers (Figure 4A), and a total of 118 DAMs were identified, including 20 upregulated and 98 down-regulated in the FB group compared to the BD group (Figure 4B, Supplementary Table S3). Based on fold-change, the top three significantly upregulated metabolites are pmb0541 (Cyanidin-3-O-(6″-O-malonyl)glucoside-5-O-glucoside), Hmjn004446 (Luteolin-7-O-(6″-caffeoyl)rhamnoside), and mws1290 (Kaempferol-3-O-(6″-p-coumaroyl)glucoside (Tiliroside)), while the top downregulated metabolites are pmb0620 (Chrysoeriol-6,8-di-C-glucoside-7-O-glucoside), mws1068 (Kaempferol), and pme2493 (Kaempferol-3,7-O-dirhamnoside (Kaempferitrin)) (Figure 4C). Moreover, compared with the early (BD) stage of the flower, mws0920 (Tricetin) and mws0355 (Catechin gallate) were significantly down-regulated in the late (FB) stages of the flower, whereas pmp001288 (Linarin) was up-regulated. Interestingly, these DAMs were predominantly enriched in the flavonoid-related pathways, such as “flavonoid biosynthesis”, “isoflavonoid biosynthesis”, and “flavone and flavonol biosynthesis” pathways (Figure 4D). Taken together, these results suggest that flavonoid metabolites changed during flower development, including some early or late-stage-specific compounds.

**Figure 5:**
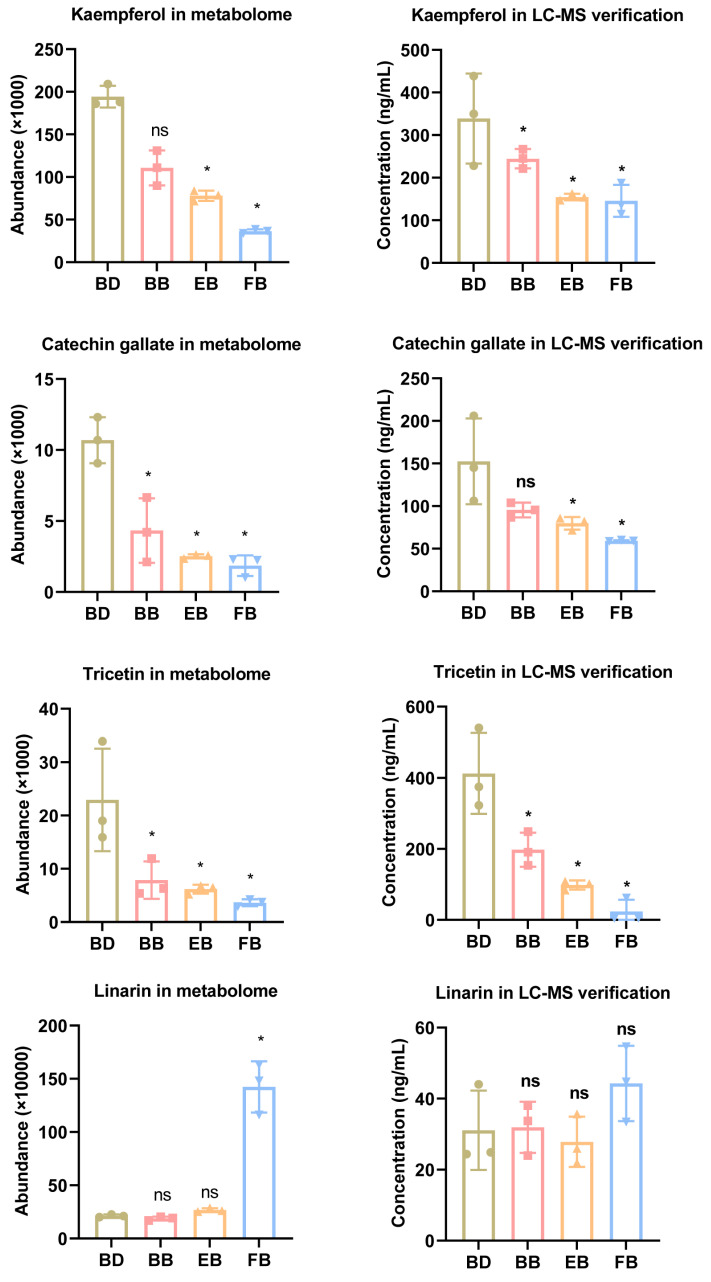
Four candidate metabolites from metabolome data were verified by LC-MS detection. ns means *p* > 0.05, * means *p* < 0.05.

To confirm the metabolome data, we selected four candidate metabolites (Kaempferol, catechin gallate, tricetin, and linarin) for LC-MS detection. As shown in Figure 5, kaempferol, catechin gallate, and tricetin were significantly down-regulated in the FB group compared with the BD group, a result consistent with the metabolomics results. These results not only emphasize the reliability of the metabolomic results but also suggest that the industrial application of these three metabolites should focus on flowers in the BD period.

### 2.6. Integrative Analyses of Transcriptome and Metabolome Reveal the Molecular Mechanism Underlying Variation in Flavonoid during Flower Development of C. morifolium

**Figure 6:**
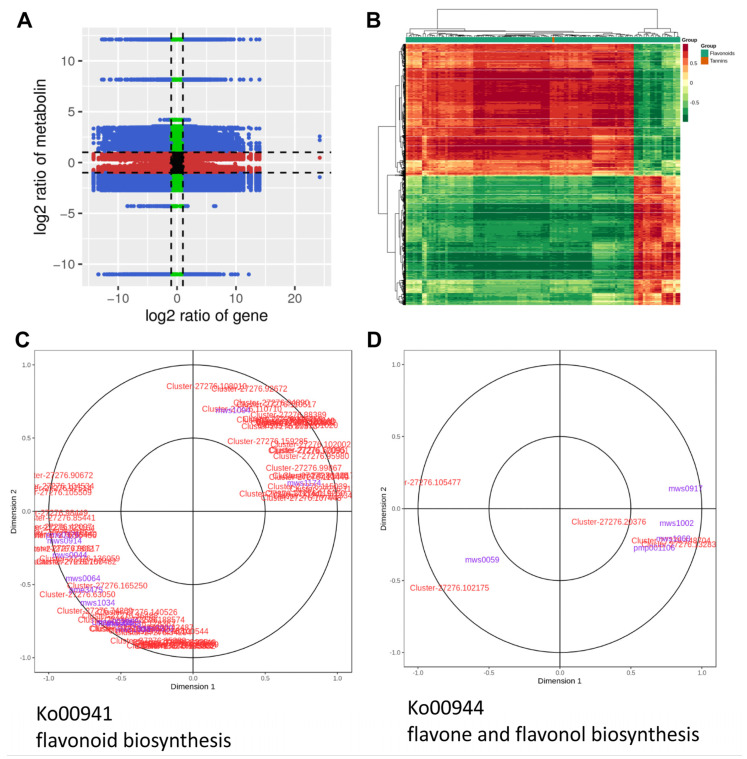
Integrative analyses of transcriptome and metabolome reveal the molecular mechanism underlying variation in flavonoids during flower development of *C*. *morifolium*. (**A**) A nine-quadrant volcano plot of Pearson correlation coefficients between genes and metabolites. (**B**) Genes and metabolites exhibiting a consistent regulatory trend were visualized in a hierarchical clustering heatmap. (**C**) The canonical correlation analysis (CCA) plot in flavonoid biosynthesis pathway. (**D**) The CCA plot in flavone and flavonol biosynthesis pathway.

To further investigate the molecular mechanisms underlying flavonoid production during the flower development of *C. morifolium*, we conducted an integrated analysis of transcriptomic and metabolomic data. Our specific focus was on elucidating the differences in flavonoid metabolism between the early (BD) and late (FB) stages of the flower. The Pearson correlation coefficients between genes and metabolites were computed using the ‘cor’ function within the R package. The correlation results were visualized in a nine-quadrant volcano plot (Figure 6A). The third and seventh quadrants in the volcano plot correspond to genes and metabolites exhibiting a consistent regulatory trend, suggesting that the changes in metabolites may be positively regulated by the genes. Genes and metabolites showing significant correlations in the third and seventh quadrants were further visualized through a hierarchical clustering heatmap (Figure 6B). We found that MDL3 was most significantly positively correlated with Kaempferol-3-O-(2″-O-acetyl)glucuronide (R = 0.959, *p* = 8.57 × 10^−7^), followed by Tricin-7-O-Glucuronide (R = 0.938, *p* = 6.69 × 10^−6^) and Quercetin-3-O-xyloside (R = 0.935, *p* = 8.1 × 10^−6^), and negatively correlated with Vitexin-2″-O-glucoside (R = −0.961, *p* = 7.02 × 10^−7^). AtADT6 was most significantly positively and negatively correlated with Cyanidin-3-O-(6″-O-malonyl)glucoside-5-O-glucoside (R = 0.908, *p* = 4.34 × 10^−5^) and Hispidulin-8-C-(2″-O-glucosyl)glucoside (R = −0.915, *p* = 3.09 × 10^−5^), respectively. ROMT was most significantly positively and negatively correlated with Kaempferol-3-O-(2″-O-acetyl)glucuronide (R = 0.964, *p* = 3.42 × 10^−7^) and Syringetin-7-O-glucoside (R = −0.971, *p* = 1.56 × 10^−7^), respectively. Interestingly, CYP81E, TPS-Cin-1, and TPS-Cin-2 were most significantly positively and negatively correlated with Kaempferol and Kaempferol-3-O-(2″-O-acetyl)glucuronide, respectively.

Furthermore, we specifically focused on the correlation between differentially expressed unigenes and DAMs within three pathways: flavonoid biosynthesis, isoflavonoid biosynthesis, and flavone and flavonol biosynthesis (Figure 4D). As depicted in the canonical correlation analysis (CCA) plot, in flavonoid biosynthesis pathway, mws1094 (Aromadendrin/Dihydrokaempferol) was significantly correlated with Cluster-27276.110710 (phlorizin synthase [EC:2.4.1.357] PGT1), and pme3475 (Butin) was significantly correlated with Cluster-27276.165250 (shikimate O-hydroxycinnamoyltransferase [EC:2.3.1.133] HCT) and Cluster-27276.63050 (trans-cinnamate 4-monooxygenase [EC:1.14.14.91] CYP73A) (Figure 6C). According to the CCA results, in the flavone and flavonol biosynthesis pathway, mws1068 (Kaempferol) and pmp001106 (Vitexin-2″-O-glucoside) were significantly correlated with Cluster-27276.148704 (isoflavone 7-O-glucoside-6″-O-malonyltransferase [EC:2.3.1.115] and IF7MAT) and Cluster-27276.132835 (flavonol-3-O-glucoside L-rhamnosyltransferase [EC:2.4.1.159], FG2) (Figure 6D). However, no correlated genes and metabolites were identified in the isoflavonoid biosynthesis pathway.

**Figure 7:**
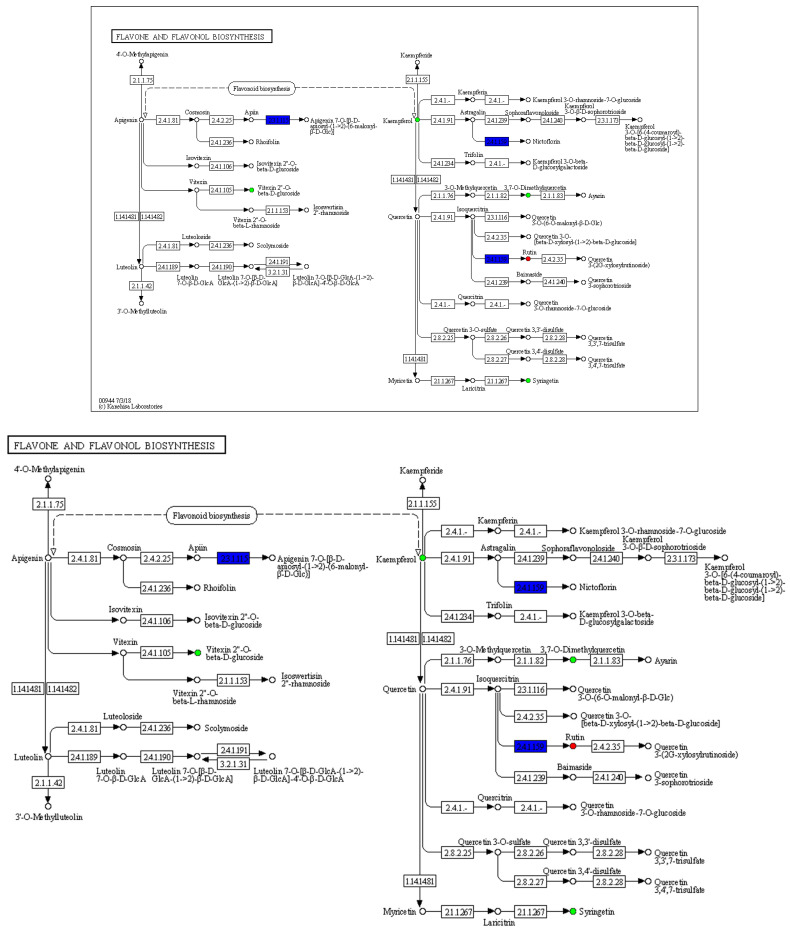
KEGG map of flavone and flavonol biosynthesis. Red and green circles indicate differentially abundance metabolites that are up-regulated and down-regulated in BD vs. FB, respectively. The blue boxes indicate genes that are up-regulated or down-regulated.

In our results, kaempferol not only exhibited the most significant differential expression but also showed the most significant correlation with multiple genes. Thus, kaempferol is drawing our attention. To elucidate the significance of kaempferol in flavonoid metabolism, we presented a KEGG map of flavone and flavonol biosynthesis pathway (Figure 7). It is evident that kaempferol and its derivatives are crucial intermediates and end products in flavonoid biosynthesis, involving aberrant expression of multiple genes during flower development.

## 3. Discussion

Flowers, serving as vital medicinal tissues of the plant *C. morifolium*, have been the focus of current research primarily on determining the flowering time and its molecular mechanisms. However, there is limited literature reporting on the molecular, metabolic regulatory network of the active component flavonoids in these flowers. In the present study, we profiled the transcriptomic landscape of the flower of *C. morifolium* at four different stages and identified many DEGs associated with flavonoid metabolism, including AtADT6, MDL3, ROMT, CYP81E, TPS-Cin-1, and TPS-Cin-2. The expression of these six genes consistently increased or decreased along the developmental trajectory of the flower. We also obtained a flavonoid-targeted metabolomics landscape of the flower at four different stages. Compared to the BD stage, 118 metabolites were differentially abundant in the FB stage, including kaempferol, catechin gallate, and tricetin. Integrative analyses demonstrated that AtADT6, MDL3, ROMT, CYP81E, TPS-Cin-1, and TPS-Cin-2 were correlated with kaempferol. This study revealed the predominant changes in flavonoid metabolism during flower maturation, with kaempferol as the primary variable, and for the first time, associated these changes with six genes.

Kaempferol is a natural flavonoid compound belonging to the flavonol class, and it is widely distributed in numerous plants, such as tea leaves, apples, grapes, and onions. It has been extensively researched and studied. The active role of kaempferol in Chrysanthemum has been reported; for example, Kanani et al. found that flavonoids (rutin, quercetin, kaempferol, and apigenin) abundance changed significantly at four flower development stages of *Rosa damascena* Mill., and found that PAL enzyme activity may be positively correlated with the yield of some flavonoid compounds [17]. The flavonoid contents were also changed with the development of Peach blossoms at six developmental stages [18]. In *Malus*, kaempferol and anthocyanin competitively regulate flower color, pollen tube growth, and seed set [19]. In addition, studies have shown that changes in compounds such as esters and ketone are also related to changes in floral fragrances at different developmental stages of *Chrysanthemum indicum* var. *aromaticum* [14]. Similar to these studies, we also found that a persistent and significant decrease in the kaempferol component occurred at different stages of flower development of *C*. *morifolium*, as well as catechin gallate and tricetin. Unfortunately, however, we do not yet know exactly what kind of flower traits are affected by regular changes in flavonoids (kaempferol, catechin gallate, and tricetin) in *C*. *morifolium*, flower color, or flower scent. But we can be sure that in applications where kaempferol and its downstream products are the active ingredients, such as flower tea or essential oil extraction, early flowers (BD group) rather than late flowers (FB group) should be collected.

In addition, the mechanism of action of kaempferol also needs to be further elucidated. In the present study, metabolomics and LC-MS together demonstrated that kaempferol, catechin gallate, and tricetin had a consistent trend, i.e., their abundance gradually decreased with flower development in *C. morifolium*. This implies that they may have some synergistic role in *C*. *morifolium* flower development. Interestingly, Wee et al. demonstrated that kaempferol can interact with apigenin and trans-cinnamic acid to inhibit xanthine oxidase in *Chrysanthemum morifolium* [20,21]. We hypothesized that there might be undiscovered forms of interactions between kaempferol, catechin gallate, and tricetin that could be involved in the regulation of flower development, and this will be part of our subsequent in-depth study.

In this study, kaempferol was proven to correlate with AtADT6, ROMT, TPS-Cin-1, TPS-Cin-2, MDL3, and CYP81E. The protein encoded by AtADT6 catalyzes the conversion of phenylalanine in the phenylpropanoid pathway in plants, generating aromatic amino acids that serve as foundational materials for the biosynthesis of anthocyanins and flavonoids [22]. However, the relationship between AtADT6 and kaempferol has not been reported, nor between MDL3 and kaempferol. ROMT is a methyltransferase primarily responsible for the 3-position hydroxyl methylation of flavones, including kaempferol, leading to the formation of the corresponding methylated products [23]. This reaction constitutes a pivotal step in the biosynthetic pathway of flavonoids, influencing the structure and biological activities of these compounds. The critical role of cytochrome P450 in secondary metabolite synthesis is well known, and CYP81E has been reported to be involved in flavonoid biosynthesis, including kaempferol [24]. TPS-Cin is postulated to be an enzyme akin to those involved in terpenoid synthesis, belonging to the terpene synthase family [25]. In certain circumstances, there may be interactions between the terpenoid biosynthetic pathway and the flavonoid metabolism pathway, particularly during a plant’s response to environmental stress [26,27]. We hypothesize that TPS-Cin might indirectly impact the biosynthesis of kaempferol by modulating the plant’s secondary metabolic pathways. This regulatory relationship could potentially occur through the sharing of substrates or intermediates or via signaling transduction networks within the plant. In short, our study supports the yet unspecified novel role of kaempferol in *C*. *morifolium* in relation to the six genes (AtADT6, ROMT, TPS-Cin-1, TPS-Cin-2, MDL3, and CYP81E).

## 4. Materials and Methods

### 4.1. Plant Material and Sample Collection

*C*. *morifolium* was grown under natural conditions in the field of Anhui Academy of Agricultural Sciences at Bozhou, Anhui province, Republic of China (33°52′ N, 115°40′ E). The annual average of temperature, sunshine, and precipitation is 14.9 °C, 2184 h, and 831 mm, respectively. Based on the previous study [7], flower samples were collected for four stages: budding (BD), bud breaking (BB), early blooming (EB), and full blooming (FB) stages. The BD stage started on Oct 28th, and the subsequent flowering phases are approximately one week later in turn. One sample was collected from six plants, and all samples were collected in triplicate from each of the sampling points. The samples were frozen in liquid nitrogen and stored at −80 °C until used.

### 4.2. RNA Isolation and Transcriptome Sequencing

RNA of 12 flower samples was isolated using a Total RNA Purification Kit (Sangon, Shanghai, China). After thawing the RNA samples on ice, the concentration, RIN/RQN, and 28S/18S values of the RNA samples were determined using the Agilent 2100 system. (Agilent, Shanghai, China) Quality-controlled RNA samples were utilized for transcriptome sequencing. In brief, total RNA samples were digested, and the temperature was optimized to denature their secondary structures. Oligo(dT) magnetic beads were employed to enrich mRNA from the digested total RNA, followed by the addition of a fragmentation reagent to fragmentize the mRNA. A pre-prepared single-strand synthesis reaction system was introduced to the fragmented mRNA, and single-stranded cDNA was synthesized on a PCR machine. Subsequently, double-stranded cDNA was synthesized. The ends of the double-stranded cDNA were repaired, an A base was added to the 3’ end, and cDNA adapters were ligated. The ligation products underwent PCR amplification, and the resulting PCR products were denatured to obtain a cDNA library. The fragment size and concentration of the library were assessed using the Agilent 2100 Bioanalyzer (Agilent, Shanghai, China). Single-stranded circular DNA molecules were replicated by rolling circle replication, forming DNA nanoballs containing over 200 copies. The obtained DNA nanoballs were loaded into the mesh holes on a high-density DNA nanochip. Sequencing was performed using the combinatorial Probe-Anchor Synthesis technology Illumina HiSeq, (Illumina, Shanghai, China), resulting in sequencing reads of 150 bp.

The image data generated by high-throughput sequencers is converted into a large quantity of high-quality data, referred to as raw data, through CASAVA base calling. Prior to conducting data analysis, it is essential to ensure that these reads exhibit sufficiently high quality to guarantee the accuracy of subsequent analyses. We rigorously implement quality control measures on the data to obtain clean reads, with the following filtering criteria: (1) Removal of reads containing adapters; (2) Elimination of paired reads when the N content in either sequencing read exceeds 10% of the total bases in that read; (3) Exclusion of paired reads when the number of low-quality bases (Q ≤ 20) in either sequencing read surpasses 50% of the total bases in that read. The clean reads were assembled using Trinity to generate unigene sequences for subsequent analysis. Following the prediction of amino acid sequences for unigene, the HMMER software was employed to align the unigene sequences with the Pfam database to obtain annotation information. The Trinity-assembled transcriptomes were used as reference sequences (ref) with the bowtie2 algorithm in RSEM to map clean reads from each sample to the reference. Normalization of the count of Mapped Reads in each sample and transcript length was performed using FPKM to determine the gene expression levels. DESeq2 was employed to filter differentially expressed unigenes based on the criteria of |log2Fold Change| ≥ 1 and FDR < 0.05. The differentially expressed unigenes were aligned against the KEGG, NR, Swiss-Prot, GO, COG/KOG, and Trembl databases using the BLAST software. The raw sequencing data have been deposited to the National Center for Biotechnology Information (NCBI) database under accession number PRJNA1138449.

### 4.3. Flavonoid-Targeted Metabolomics Analysis

#### 4.3.1. Flower Sample Extraction

The flower samples were placed in a freeze dryer (Scientz-100F, Scientz, Ningbo, China) for vacuum freeze-drying. Subsequently, the dried samples were ground into a powder using a ball mill (MM 400, Retsch, Shanghai, China) at a frequency of 30 Hz for 1.5 min. Approximately 100 mg of the resulting powder was weighed and dissolved in 1.2 mL of 70% methanol extraction solution. The sample was vortexed every 30 min for 30 s, totaling six vortexing cycles, and then stored overnight in a 4 °C refrigerator. After centrifugation at 12,000 rpm for 10 min, the supernatant was aspirated, and the sample was filtered through a microporous membrane (0.22 μm pore size) and stored in a sample vial for subsequent UPLC-MS/MS analysis.

#### 4.3.2. UPLC Conditions

The data acquisition instrument system primarily comprises Ultra Performance Liquid Chromatography (UPLC) using the SHIMADZU Nexera X2 instrument (https://www.shimadzu.com.cn/) on November 21, 2020 and Tandem Mass Spectrometry (MS/MS) utilizing the Applied Biosystems 4500 QTRAP instrument (http://www.appliedbiosystems.com.cn/) on 21 November 2020.

The chromatographic column employed was an Agilent SB-C18 (1.8 µm, 2.1 mm × 100 mm, Agilent, Shanghai, China). The mobile phase consisted of ultra-pure water with 0.1% formic acid as phase A and acetonitrile with 0.1% formic acid as phase B. The elution gradient started with a 5% proportion of phase B at 0.00 min, increased linearly to 95% within 9.00 min, maintained at 95% for 1 min, decreased to 5% from 10.00 to 11.10 min, and finally equilibrated at 5% until 14 min. The flow rate was set at 0.35 mL/min, the column temperature at 40 °C, and the injection volume at 4 μL.

#### 4.3.3. ESI-Q TRAP-MS/MS

Linear Ion Trap and Triple Quadrupole (QQQ) scans were obtained on the Triple Quadrupole Linear Ion Trap Mass Spectrometer (Q TRAP), SCIEX AB4500 Q TRAP UPLC/MS/MS system. This system was equipped with an ESI Turbo Ion Spray interface and was operated in both positive and negative ion modes using Analyst 1.6.3 software (AB Sciex). The ESI source operating parameters were as follows: ion source was turbo spray; source temperature was 550 °C; ion spray voltage (IS) set at 5500 V (positive ion mode)/−4500 V (negative ion mode); ion source gas I (GSI), gas II (GSII), and curtain gas (CUR) were set to 50, 60, and 25.0 psi, respectively, and collision-induced ionization parameters were set to high. Instrument tuning and mass calibration were performed using 10 and 100 μmol/L polyethylene glycol solutions in QQQ and Linear Ion Trap modes, respectively. QQQ scans utilized the MRM mode, with collision gas (nitrogen) set to medium. The optimization of DP and CE for each MRM ion pair was accomplished through further DP and CE optimization. Specific sets of MRM ion pairs were monitored for each period based on the elution of metabolites during that period. Based on the in-house MWDB (MetWare database), a qualitative analysis of the substance was performed using tandem mass spectrometry information. During the analysis, isotope signals, duplicate signals containing K^+^ ions, Na^+^ ions, NH4^+^ ions, and signals duplicating fragment ions originating from larger molecules were excluded. Metabolite quantification was accomplished using the Multiple Reaction Monitoring (MRM) mode of the Triple Quadrupole Mass Spectrometry using Analyst 1.6.3 software. Significantly differentially abundant metabolites (DAMs) between groups were determined by VIP ≥ 1 and absolute Log2FC (fold change) ≥1 or ≤0.5. VIP values were extracted from OPLS-DA results, which also contain score plots and permutation plots, and were generated using the R package MetaboAnalystR 1.0.1. The data was log transform (log2) and mean centering before OPLS-DA. To avoid overfitting, a permutation test (200 permutations) was performed.

### 4.4. Integrated Analysis of Transcriptomic and Metabolomic Data

The integrated analysis of transcriptomics and metabolomics was conducted by Wuhan Biomarker Technologies Co., Ltd. The analysis encompassed the normalization and statistical analysis of bulk data from transcriptomics and metabolomics. The analysis involved establishing relationships among data from two molecular levels and performing functional analysis, metabolic pathway enrichment, and correlation analysis.

### 4.5. Metabolite Confirmation Using LC-MS

Approximately 100 mg of flower samples were ground into a powder and dissolved in 1 mL absolute ethyl alcohol, followed by ultrasonic treatment on ice for 1 h. After centrifugation at 17,000 g for 15 min, the supernatant was filtered through a microporous membrane (0.22 μm pore size) and stored. The blank matrix was used as a diluent for the preparation of standard curves, and the standards were diluted to specific concentrations for use in making standard curves.

The HCLASS instrument (Waters, Milford, MA, USA) and AB4500 Q TRAP were applied for LC-MS. The chromatographic column employed was a ZORBAX Rx-C8 (1.8 µm, 4.6 mm × 10 mm, Agilent, Shanghai, China). The mobile phase consisted of ultra-pure water with 0.1% formic acid as phase A and acetonitrile as phase B. The elution gradient started with a 5% proportion of phase B at 0.00 min, maintained at 5% for 1 min, increased linearly to 20% within 5.00 min, increased to 40% within 10 min, increased to 100% from 10.00 to 15.00 min, maintained at 100% for 1 min, increased to 5% from 16.00 to 16.20 min, and finally equilibrated at 5% until 18 min. The flow rate was set at 400 µL/min, the column temperature at 40 °C, and the injection volume at 1 μL. QQQ scans utilized the MRM mode in ESI-positive ion modes. The ESI source operating parameters were as follows: source temperature was 500 °C; curtain gas and collision gas were set to 25 and 10 psi, respectively; ion spray voltage (IS) was set at 4500 V. The sample concentration was calculated using the internal standard method with standard and standard curves using MultiQuant 3.0.3 analysis software.

## 5. Conclusions

The present study profiled transcriptome and metabolome at four stages of the flower of *C*. *morifolium* and revealed a novel regulatory relationship between six key genes (AtADT6, MDL3, ROMT, CYP81E, TPS-Cin-1, and TPS-Cin-2) and kaempferol in flavonoid metabolism. Our findings provide a new idea for interfering with flavonoid production, especially Kaempferol, in flowers of *C*. *morifolium*.

## Data Availability

The raw sequencing data have been deposited to the National Center for Biotechnology Information (NCBI) database under accession number PRJNA1138449.

## Supplementary Materials

The following supporting information can be downloaded at: https://www.mdpi.com/article/10.3390/ijms251910261/s1.
