# Supplementary material for: Delineating Molecular Regulatory of Flavonoids Indicated by Transcriptomic and Metabolomics Analysis during Flower Development in Chrysanthemum morifolium ‘Boju’
Source: Int J Mol Sci. 2024 Sep 24;25(19):10261. doi: 10.3390/ijms251910261 (PMC11476272; doi:10.3390/ijms251910261)
Supplement: Supplementary file 1 [file ijms-25-10261-s001.zip › Supplemental Table S1.pdf]

Supplemental Table S1. Quality control of transcriptome data.

Sample	Raw Reads	Clean Reads	Clean Base (G)	Error Rate (%)	Q20(%)	Q30 (%)	GC Content(%)
BD-1	68472108	59034778	8.86	0.03	97	92.33	42.07
BD-2	67000106	60628082	9.09	0.03	96.83	91.85	42.05
BD-3	62425888	56871022	8.53	0.03	96.81	91.82	42.12
BB-1	61680754	56520440	8.48	0.03	96.78	91.76	42.05
BB-2	63640782	58983798	8.85	0.03	96.94	92.16	42.13
BB-3	52547150	49078780	7.36	0.03	96.64	91.4	42.12
EB-1	65945748	58104204	8.72	0.03	96.86	91.98	42.18
EB-2	62113164	56934748	8.54	0.03	97.03	92.38	42.1
EB-3	64836768	59635156	8.95	0.03	96.78	91.77	42.08
FB-1	66522946	56733334	8.51	0.03	96.73	92.09	41.09
FB-2	59718956	54885392	8.23	0.03	96.71	91.58	42.05
FB-3	63560114	58268064	8.74	0.03	96.61	91.35	42.14
