# Supplementary material for: Delineating Molecular Regulatory of Flavonoids Indicated by Transcriptomic and Metabolomics Analysis during Flower Development in Chrysanthemum morifolium ‘Boju’
Source: Int J Mol Sci. 2024 Sep 24;25(19):10261. doi: 10.3390/ijms251910261 (PMC11476272; doi:10.3390/ijms251910261)
Supplement: Supplementary file 1 [file ijms-25-10261-s001.zip › Supplemental Table S2.pdf]

Supplemental Table S2. Summary for the transcriptome assembly.

Type	Number	Mean Length	N50	N90	Total Bases
Transcript	796142	603	747	294	4.8E+08
Unigene	223237	648	824	315	1.45E+08
