# Supplementary figures and images for: Delineating Molecular Regulatory of Flavonoids Indicated by Transcriptomic and Metabolomics Analysis during Flower Development in Chrysanthemum morifolium ‘Boju’
Source: Int J Mol Sci. 2024 Sep 24;25(19):10261. doi: 10.3390/ijms251910261 (PMC11476272; doi:10.3390/ijms251910261)

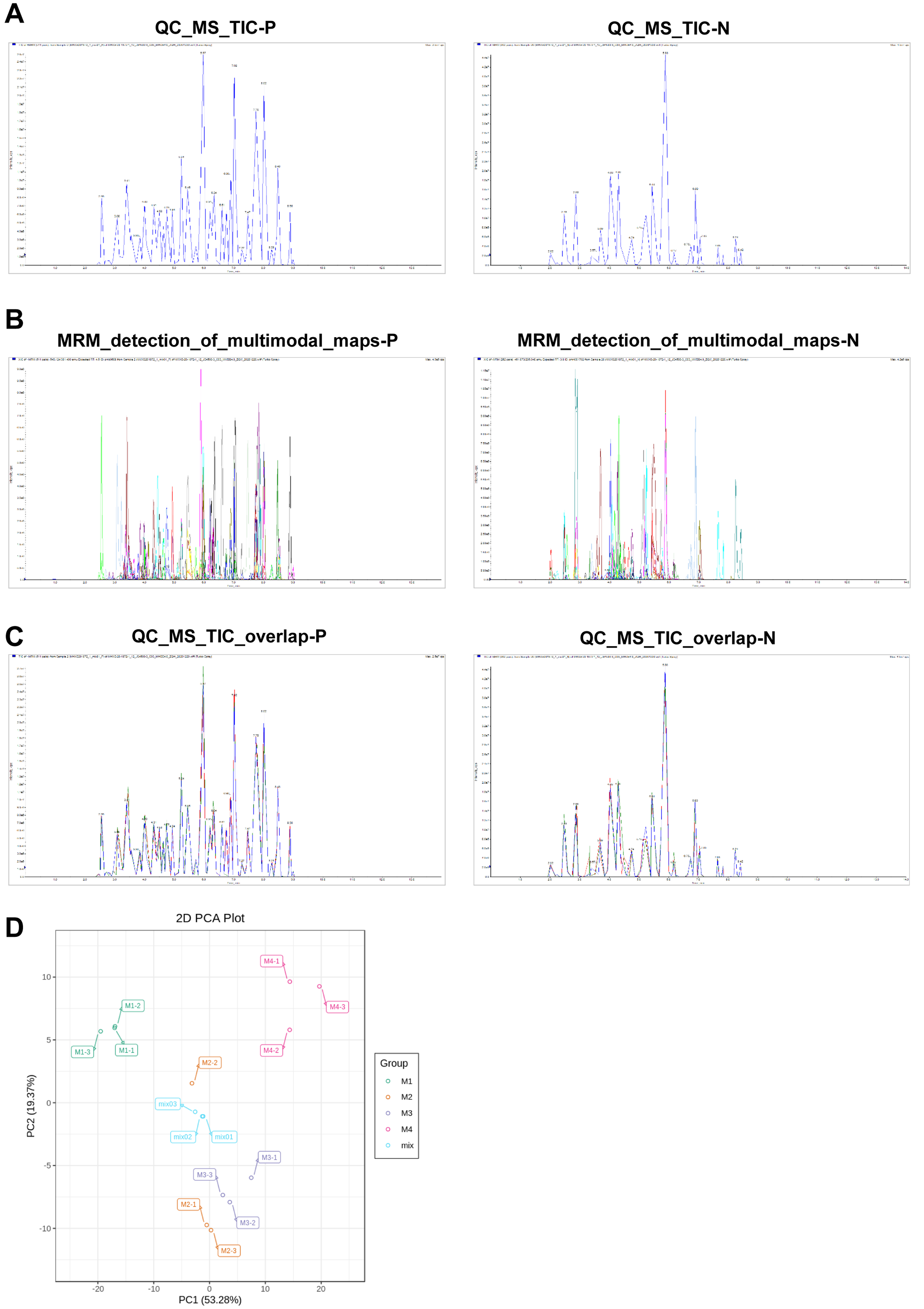

Supplement: Supplementary file 1 [file ijms-25-10261-s001.zip › Supplemantal Figure S1.tif]

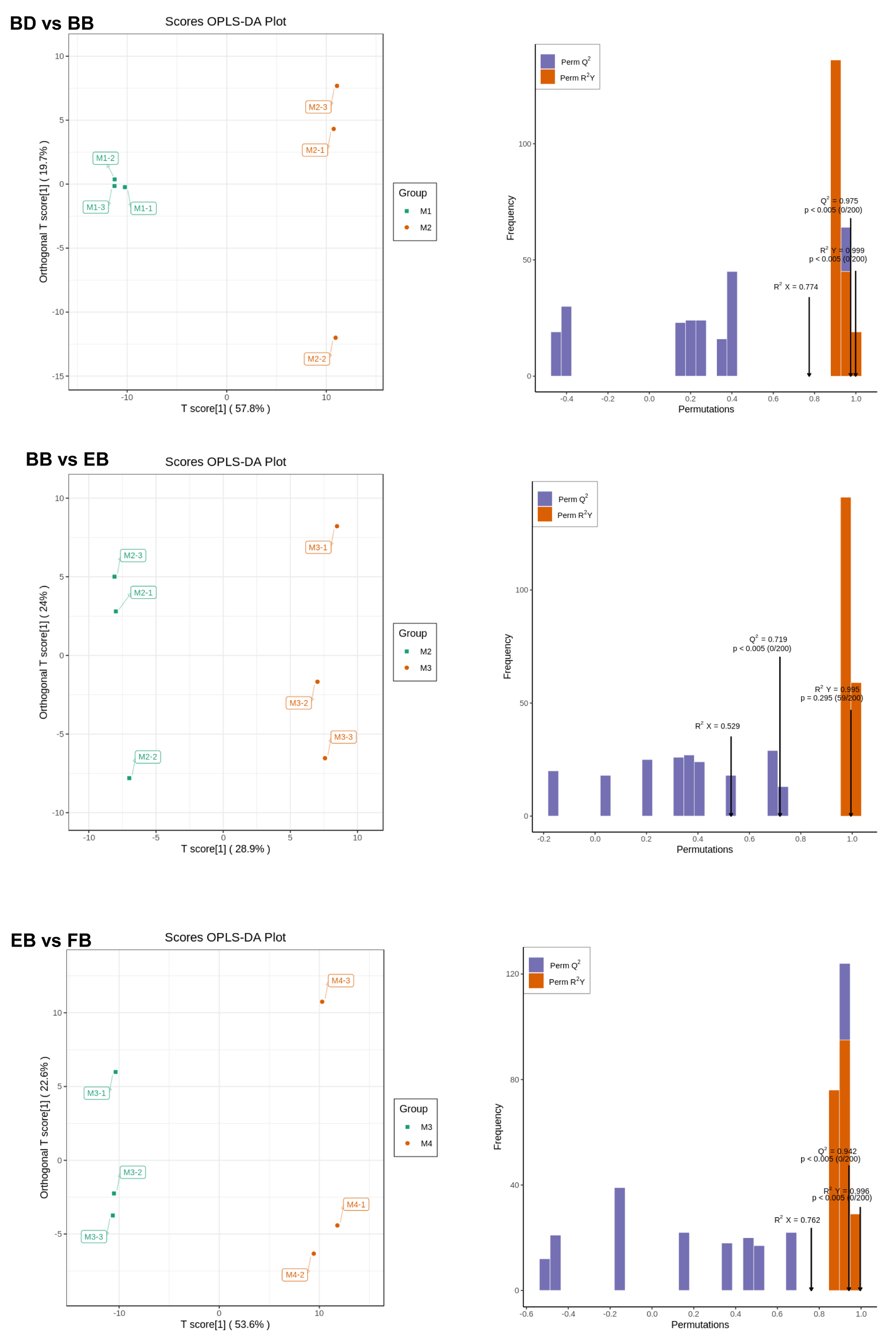

Supplement: Supplementary file 1 [file ijms-25-10261-s001.zip › Supplemental Figure S2.tif]
